# Postnatal dysregulation of Notch signal disrupts dendrite development of adult-born neurons in the hippocampus and contributes to memory impairment

**DOI:** 10.1038/srep25780

**Published:** 2016-05-13

**Authors:** Xue-Feng Ding, Xiang Gao, Xin-Chun Ding, Ming Fan, Jinhui Chen

**Affiliations:** 1 Spinal Cord and Brain Injury Research Group, Stark Neuroscience Research Institute, and Department of Neurological Surgery, Indiana University School of Medicine, Indianapolis, IN 46202, USA; 2 Department of Cognitive sciences, Beijing Institute of Basic Medical Sciences, Beijing 100850, P. R. China; 3 Department of Pathology, Indiana University School of Medicine, Indianapolis, IN 46202, USA

## Abstract

Deficits in the Notch pathway are involved in a number of neurologic diseases associated with mental retardation or/and dementia. The mechanisms by which Notch dysregulation are associated with mental retardation and dementia are poorly understood. We found that Notch1 is highly expressed in the adult-born immature neurons in the hippocampus of mice. Retrovirus mediated knockout of *notch1* in single adult-born immature neurons decreases mTOR signaling and compromises their dendrite morphogenesis. In contrast, overexpression of Notch1 intracellular domain (NICD), to constitutively activate Notch signaling in single adult-born immature neurons, promotes mTOR signaling and increases their dendrite arborization. Using a unique genetic approach to conditionally and selectively knockout *notch 1* in the postnatally born immature neurons in the hippocampus decreases mTOR signaling, compromises their dendrite morphogenesis, and impairs spatial learning and memory. Conditional overexpression of *NICD* in the postnatally born immature neurons in the hippocampus increases mTOR signaling and promotes dendrite arborization. These data indicate that Notch signaling plays a critical role in dendrite development of immature neurons in the postnatal brain, and dysregulation of Notch signaling in the postnatally born neurons disrupts their development and thus contributes to the cognitive deficits associated with neurological diseases.

The Notch signaling pathway is one of the most conserved pathways among species that shows versatility in its functions^1^,^2^. Activation of Notch receptors (Notch1, Notch2, Notch3, and Notch4 in mammals and Notch in *Drosophila*) by ligands (Jagged1, Jagged2, Delta1, Delta-like 1 [Dll1], Dll3, and Dll4 in mammals or Delta and Serrate in *Drosophila*) initiates the proteolytic processing events by a presenilin-dependent protease to release the Notch intracellular domain (NICD)^3^,^4^,^5^,^6^. NICD subsequently translocates to the nucleus, and activates the major downstream nuclear target for Notch.

Presenilins are essential components of the gamma-secretase complex. In humans, presenilins are the first genes identified to associate with early onset forms of familial Alzheimer’s disease^7^. Dysregulation of the Notch signaling pathway has been shown to contribute to a number of neurological diseases including Alzheimer’s disease (AD)^8^, cerebral autosomal dominant arteriopathy with subcortical infarcts and leukoencephalopathy (CADASIL)^9^, and cortical dysplasia^10^. However, how Notch dysregulation is related to these neurological disorders is still poorly understood.

During early neural development, Notch is highly expressed in the neural precursor cells and plays a critical role in regulating neural proliferation and differentiation^11^. Besides its critical functions in a variety of cell-fate decisions during development^11^,^12^, the Notch pathway expresses in the postnatal brain including the neurogenic regions of the adult brain^13^, the subventricular zone (SVZ) and the subgranular zone (SGZ) where new neurons continue to be generated throughout the life span of adult mammals.

A larger number of neurons in the hippocampal dentate gyrus are generated postnatally^14^,^15^. In rodents, 15% of granule cells are generated before birth; the rest of the granule cells are generated after birth, most in the first week after birth, and last up to one month before reduced to a very low level of neurogenesis in the dentate gyrus through adulthood. New neurons are continuously generated in the SGZ of the dentate gyrus beyond development and into adulthood^16^,^17^,^18^,^19^,^20^,^21^. These postnatally born granular neurons are electrically active^22^,^23^,^24^,^25^,^26^,^27^, are capable of firing action potentials and receiving synaptic inputs^25^,^26^,^27^, and may contribute to specific brain functions, such as learning, memory, and mood modulation^28^,^29^.

Adult neurogenesis represents a striking example of structural plasticity in the mature CNS that may be compromised by disease or injury. We found that Notch1 is highly expressed in the postnatally born immature neurons in the hippocampus. We previously reported a unique genetic model that allows us to conditionally manipulate a targeted gene in the postnatally born immature neurons in the hippocampus^30^,^31^. Our results showed that inactivation or activation of Notch1 signaling pathway in postnatally born immature neurons in the hippocampus dramatically affects their morphological development and impairs the cognitive functions, which may contribute to neurological diseases such as Alzheimer’s disease.

## Materials and Methods

### Animal care

Mice were housed under a 12-hour light/dark cycle with food and water available. The Pro-opiomelanocortin (POMC)-Cre mouse line^32^ was kindly provided by Dr. Lowell at Beth Israel Deaconess Medical Center. Notch1^flox/flox^ mice were created by Raphael Kopan at Washington University in St. Louis^33^. Flox-stop-flox-NICD mice were provided by Jie Shen at Harvard Medical School^34^, in which *NICD* expression is under the control of the chicken *β-actin* promoter and a floxed transcriptional and translational “stop” cassette. The *notch1* conditional knockout (Notch1-cKO) mice were generated by mating *notch1* floxed mice with POMC-Cre mice. *NICD* over-expression mice were generated by mating *NICD* floxed mice with POMC-Cre mice (NICD-cOE). All the procedures for using these animals were according to the principles outlined in “Guidelines for Care and Use of Experimental Animals” and approved by the Institutional Animal Care and Use Committees of Indiana University School of Medicine.

### Retrovirus-mediated gene manipulation in single cells

Retrovirus carrying green fluorescent protein (GFP)-Cre was used to excise the floxed *notch1* (Ayumu Tashiro *et al*., 2007). Retrovirus carrying the Ds-Red fluorescent protein (RFP) was used to indicate the dendrite morphology of newborn neurons. The above two kinds of retrovirus (gift from Dr. Fred H. Gage, Salk Institute) were mixed (1:1) and injected into the mouse hilus in the dentate gyrus of the hippocampus using a Hamilton syringe. The floxed *notch1* in the newborn neurons infected by both retroviruses simultaneously will be knocked out by GFP-Cre and the morphology changes of dendrites can be observed via Ds-Red. A similar approach was used to excise the floxed stop code in front of *NICD*. NICD will be over-expressed in the newborn neurons infected by retrovirus expressing GFP-Cre, and the dendrites will be observed via Ds-Red as well.

### Immunocytochemistry

Animals were deeply anesthetized with Avertin, and then were perfused transcardially with cold 0.9% heparinized saline followed by fixative containing 4% paraformaldehyde (PFA) in phosphate buffered saline (PBS). Brains were post-fixed overnight and cryoprotected with 30% sucrose for 48 hours. Serial coronal sections (30 μm for immunofluorescence [IF] or 60 μm for morphology analysis) were cut using a cryostat (Leica CM1950) and stored at −20 °C. Immunofluorescence was carried out as follows: Sections were rinsed in PBS 3 times and incubated in blocking solution (0.1% Triton X-100, 1% bovine serum albumin, and 5% normal goat serum in PBS) for 1 hour at room temperature, followed by an overnight incubation with primary antibody at 4 °C. Sections were then washed 3 times with PBS and incubated with the secondary antibody at room temperature for 2 hours. After being treated with 4′,6-diamidino-2-phenylindole (DAPI) for 2 minutes, sections were washed with PBS 3 times and mounted on the slides. When sections dried, the Fluoromount-G was applied for preserving the fluorescence. Primary antibodies and their final concentrations were as follows: anti-Notch1 antibody (1:100, mouse, Sigma), anti-Dcx antibody (1:1000, guinea pig, Millipore), anti-RFP antibody (1:1000, rabbit, Invitrogen), anti-GFP antibody (1:1000, chicken, Abcam), anti-pS6 antibody (1:200, rabbit, Cell signaling). Secondary antibodies from Jackson ImmunoResearch Laboratories Inc. were all applied at the same dilution of 1:1000. Cresyl violet was used for counter-staining.

### Dendrite quantification

For analyzing dendritic morphologies of RFP or RFP/GFP-positive neurons, neurons (n > 20) from 3 mice were sampled. The microscope settings were chosen so that the RFP fluorescence was within the brightness range required to clearly visualize the entire cell, and they were maintained for all experiments in the same category. Series of z-stack images were collected, encompassing all neuronal processes for reconstructing the 3-dimensional (3D) image of each cell using Neurolucida. Total number of branches and total neurite length were determined. For tracing dendrite arbors by doublecortin (Dcx) staining in genetically modified mice, first, the Dcx-positive cell bodies in the granular cell layer (GCL)/SGZ were counted and then the number of Dcx-positive dendrites in the molecular layer (ML) was measured. The branch number of Dcx-positive cells was expressed as ratio of Dcx-positive dendrites to Dcx-positive cell bodies.

### Morris water maze test

Morris water maze (MWM) test was used to assess the function of mouse spatial learning and memory (Charles V Vorhees and Michael T Williams, 2006), which had been carried out and recorded by TopScan software (version 3). The test lasted 6 days, including a 5-day trial of learning and a 1-day trial of memory. During the learning days, mice underwent 4 trials from different start positions. Mice were released in cold water at 18 °C. Each mouse had 1 minute to find the platform hidden 1 cm underwater. Once a mouse reached the platform, it was allowed to remain 15 seconds to allow it to build memories. If a mouse didn’t find the platform within 1 minute, then the experimenter put it on the platform and let it remain for 15 seconds. During a 1-minute rest between trials, mice stayed in a dry cage with a towel and a warm light. On the 6^th^ day, the platform was removed and each mouse started from a new position. Only one trial was performed on the last day. Latency was recorded to analyze the function of learning and memory.

### Statistical Analysis

The data on dendrite morphology were analyzed using unpaired and 2-tailed Student’s t-test, while the data on learning and memory was analyzed using repeated measures ANOVA followed by LSD as a post hoc test.

## Results

### Notch 1 was highly expressed in the adult born immature neurons in the hippocampus

**Figure 1:**
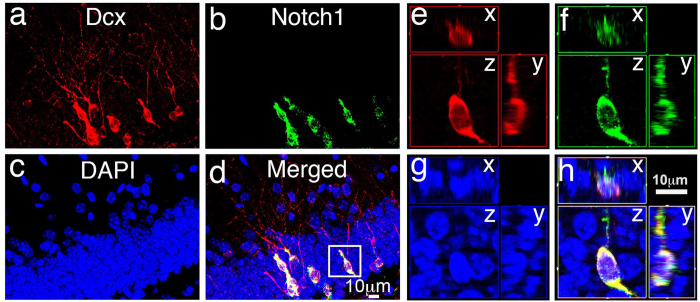
Notch1 expressed in the adult-born immature neurons in the dentate gyrus (DG). (**a**) Doublecortin (Dcx) staining in DG (red). (**b**) Notch1 staining in DG (green). (**c**) DAPI staining show the granular cell layer/subgranular zone (GCL/SGZ) structure. (**d**) Merged image of (**a–c**). (**e–h**) 3-dimensional image of single Dcx-positive cell (red, from white box in **d**) is co-labeled with Notch1 (green).

Immature neurons are continuously generated in the adult hippocampus. Double immunostaining with antibodies against doublecortin (Dcx) and Notch 1 was performed to assess the expression of Notch1 in the adult hippocampus. Dcx is a widely used cell marker for immature neurons^35^,^36^. Notch1 was detectable in the Dcx-positive adult-born immature neurons in the hippocampus at 12 weeks of age (Fig. 1a–d). Images of 3D reconstruction confirmed Notch1 expression in the adult-born immature neurons (Fig. 1e–h). Notch1 was not detectable in the mature neurons in the hippocampus with immunostaining, although its mRNA expression was detectable in the mature neurons^37^,^38^,^39^. These data indicated that Notch 1 is highly expressed in the adult-born immature neurons.

### Retrovirus-mediated single cell knockout of *notch1* in the adult-born immature neurons impaired their dendrite morphogenesis

To investigate the role of Notch1 signal in the adult-born immature neurons in the hippocampus, we first conditionally knocked out *notch1* from them using a retrovirus-mediated Cre recombinase expression approach. *notch1* floxed mice received stereotaxical injection of a mixture of two retroviruses expressing RFP or expressing Cre recombinase and GFP fusion protein in the hippocampus. Retroviruses will infect the dividing neural precursor cells (NPCs) in the hippocampus and integrate into their chromosomes leading to consistent expression of the carried genes. This approach conditionally knockouts the *notch1* in the virus-infected cells in which Cre recombinase will be expressed. At the same time, the expression of GFP or RFP also labels the birthday of the infected cells allowing us to trace the fate of the infected NPCs and their progenies, immature neurons. Furthermore, the expression of RFP will exhibit the morphology of the neurons allowing us to quantify their dendrite morphogenesis.

**Figure 2:**
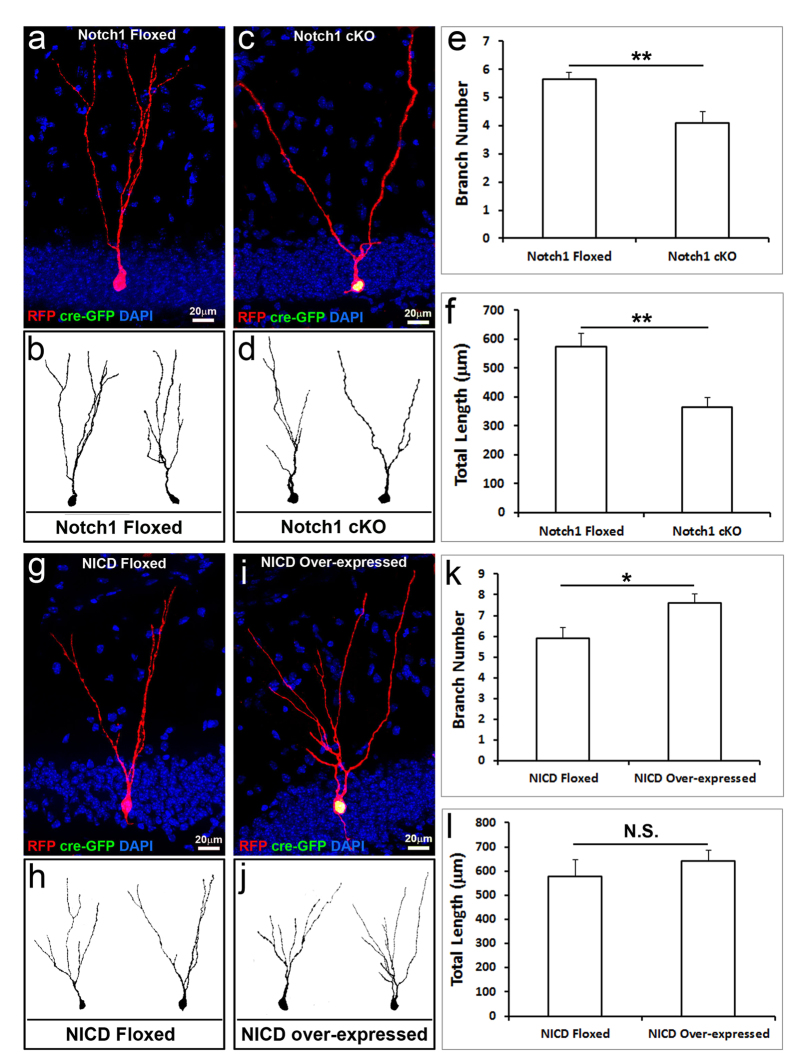
Retrovirus-mediated single-cell knocked out Notch1 or over-expressed Notch intracellular domain (NICD) in newborn neurons of adult mouse hippocampus. (**a**) 3 weeks after retrovirus infection, newborn neurons with only red fluorescent protein (RFP) expressing in *notch1* floxed mouse hippocampus (red, considering as normal control) exhibited typical neuronal structure with the cell body located at the inner granular cell layer (GCL) and the dendrite tree extended toward the molecular layer (ML). (**b**) Neurolucida reconstructed representative control neurons. (**c**) 3 weeks after retrovirus infection, Notch1 knocked out newborn neurons; both RFP (red) and Cre-GFP (green, in nucleus) expressing neurons showed fewer dendrite branches. (**d**) Neurolucida reconstructed representative Notch1 knocked out neurons. (**e**) The quantification data of dendrite branch number in control or Notch1 knocked out newborn neurons. (**f**) The quantified dendrite total length of control or Notch1 knocked out newborn neurons. (**g**) 3 weeks after retrovirus infection, newborn neurons with only RFP expressing in *NICD* floxed mouse hippocampus (red, considering as normal control) exhibited typical neuronal structure with the cell body located at the inner granular cell layer (GCL) and the dendrite tree extended toward the molecular layer (ML). (**h**) Neurolucida reconstructed representative control neurons. (**i**) 3 weeks after retrovirus infection, NICD over-expressed newborn neurons with both RFP (red) and Cre-GFP (green, in nuclear) expressing showed the increasing of dendrite branches and complexity. (**j**) Neurolucida reconstructed representative NICD over-expressed neurons. (**k**) The quantification data of dendrite branch number in control or NICD over-expressed newborn neurons. (**l**) The quantified dendrite total length of control or NICD overexpressed newborn neurons. (n > 20 for each group, *p < 0.05, **p < 0.01, N.S.: not significant).

Twenty-one days after the viral injection, mice were sacrificed to assess dendrites of immature neurons with RFP or/and GFP expression in the hippocampus. Cells expressing RFP were also co-labeled by doublecortin (Dcx), indicating that those RFP-positive cells were immature neurons in the adult brain (Supplemental Fig. 1). These RFP-positive but Cre/GFP fusion protein-negative cells were wild-type adult-born immature neurons (Supplemental Fig. 1a and Fig. 2a). These adult-born immature neurons generated 21 days earlier exhibited typical neuronal morphology with dendrites oriented towards the ML (Fig. 2a). RFP expression in adult-born immature neurons includes their dendrites, thus allowing us to reconstruct and quantify their dendrite morphology (Fig. 2b). Cells expressing both RFP and GFP were *notch1* knock-out adult-born immature neurons (Fig. 2c,d), in which RFP was expressed in neurons and their processes, while GFP located in the nucleus due to its fusion with Cre recombinase. Cre recombinase includes a nucleus translocation signal. The wild-type immature neurons at 21 days after viral injection had an average of 5.7 dendrite branches (Fig. 2e); the average total length of dendrites in each immature neuron was 573.3 μm (Fig. 2f). In the Notch1 mutant immature neurons, the average number of dendrite branches reduced to 4.1 (p < 0.01) (Fig. 2e), while the average total length of dendrites in each immature neuron reduced to 364.1 μm (p < 0.01) (Fig. 2f). These results indicated that *notch1* knockout impairs dendrite development by reducing their number of branches and total length.

We next assessed the dendrite development of adult-born immature neurons by overexpressing *NICD* to constitutively activate the Notch signaling pathway using the same retrovirus approach. The wild-type immature neurons in the hippocampus of *NICD* floxed mice at 21 days after viral injection (Fig. 2g,h) had an average of 5.9 dendrite branches and the average total length of dendrites in each immature neuron was 579.3 μm (Fig. 2k,l). NICD overexpression (Fig. 2i,j) significantly increased the average number of dendrite branches to 7.6, an increase of 28.8% (p < 0.05) (Fig. 2k,l), while the average of total dendrite length in each immature neuron slightly increased to 643.5 μm (p > 0.05). Together, these data showed that overactivation of Notch signaling pathway in the adult-born neurons significantly increased their number of dendrite branches.

### Conditional knockout of Notch1 in the adult-born immature neurons in the adult hippocampus reduced the number of dendrite branches

Global deletion of *notch1* by conventional gene knockout techniques leads to embryonic lethality^40^,^41^,^42^,^43^,^44^,^45^,^46^. To further determine the function of Notch1 for development of immature neurons in the postnatal and adult hippocampal dentate gyrus (DG), we deleted the *notch1* gene from postnatal-born immature neurons with a temporal, regional, and cell-type restricted manner using a cre-floxp conditional knockout system. In our previous study, we characterized a transgenic mouse strain, in which Cre recombinase, driven by the pro-opiomalanocortin (POMC) promoter, was expressed in the newborn immature neurons of the hippocampal dentate gyrus from postnatal day 5 (P5) onward^32^,^47^. The Cre will mediate the targeted gene deletion selectively in the immature neurons right after they exit the last cell cycle from postnatal day 5 (P5) onward without affecting embryonic development of the hippocampus.

**Figure 3:**
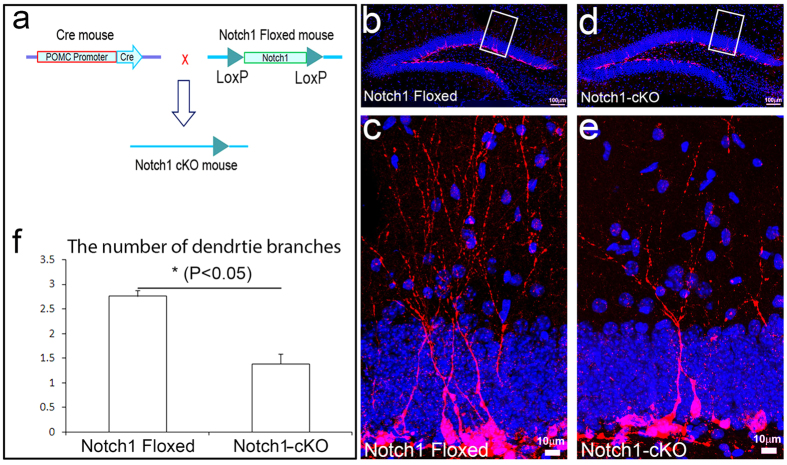
Loss of Notch1 significantly decreased the dendritic arborization. (**a**) Schematic of generating the Cre-mediated *notch1* conditional knockout (Notch1-cKO) mice. The *notch1* exon will be excised by Cre recombinase. (**b**) Doublecortin (Dcx) staining (red) showed the newborn immature neurons in *Notch1*-floxed mouse hippocampus (control). (**c**) High power image of white box in (**b**). The Dcx-positive cells exhibited typical neuronal structure with the cell body located at the inner granular cell layer (GCL) and the dendrite tree extended toward the molecular layer (ML). (**d**) Dcx staining (red) showed the newborn immature neurons in Notch1-cKO mouse hippocampus. (**e**) High power image of white box in (**d**). The Dcx-positive cells in Notch1-cKO mouse showed the decreasing of dendritic arborization compared to the control (**c**). (**f**) The quantified dendrite branch number of Dcx-positive immature neurons in floxed or Notch1-cKO mice. (4 sections from each of 3 mice per group in comparable position were used for counting. *p < 0.05).

**Figure 4:**
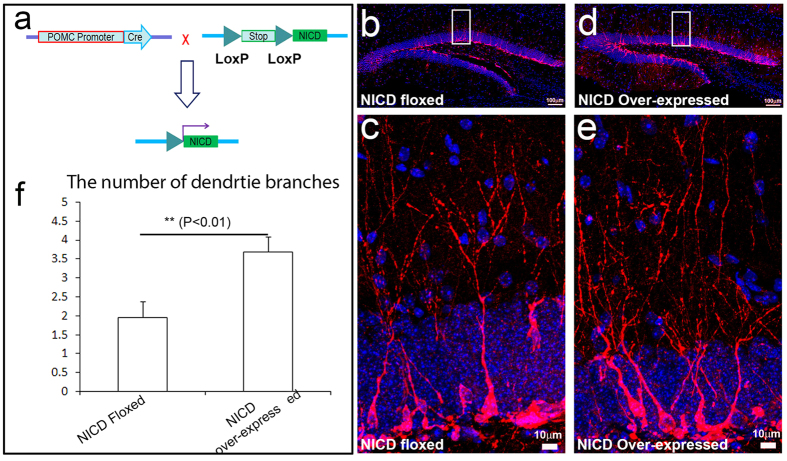
Notch intracellular domain (NICD) over-expression significantly promotes the dendritic arborization. (**a**) Schematic of generating the Cre-mediated NICD over-expressed mice. The stop codon in front of *NICD* will be excised by Cre and NICD will express in newborn neurons continuously afterward. (**b**) Doublecortin (Dcx) staining (red) showed the newborn immature neurons in *NICD* floxed adult mouse hippocampus. (**c**) High power image of white box in (**b**). The Dcx-positive cells exhibited typical neuronal structure with the cell body located at the inner granular cell layer (GCL) and the dendrite tree extended toward the molecular layer (ML). (**d**) Dcx staining (red) showed the newborn immature neurons in NICD conditional overexpression (NICD-cOE) adult mouse hippocampus. (**e**) High power image of white box in (**d**). The Dcx-positive cells in a NICD-cOE mouse showed the significant increase of dendritic arborization compared to the control (**c**). (**f**) The quantified dendrite branch number of Dcx-positive immature neurons in floxed or NICD-cOE mice. (4 sections from each of 3 mice per group in comparable position were used for counting. **p < 0.01).

The POMC-Cre transgenic mice were crossed with mice carrying the floxed *notch1* allele (Notch1^flox/flox^) to generate POMC-Cre::Notch1^flox/flox^ conditional knockout (cKO) mice (Fig. 3a). Although the expression of Notch1 in the immature neurons is detectable in the control mice using immunostaining (Fig. 1), it was undetectable in the adult-born immature neurons in the Notch1-cKO mice with double immunostaining (Supplemental Fig. 2). Meanwhile, the POMC-Cre transgenic mice were crossed with mice carrying the floxed-stop codon-floxed-NICD to generate POMC-Cre::^flox^Stop codon^flox^-NICD conditional overexpression (cOE) mice (Fig. 4a). Double immunostaining showed that NICD was very highly expressed not only in the adult-born immature neurons, but also in the granular neurons in the dentate gyrus (Supplemental Fig. 3). In rodents, 15% of granule cells are generated before birth; the rest of the granule cells are generated after birth, most in the first week after birth, and then last up to one month before reducing to a very low level of neurogenesis in the dentate gyrus through adulthood. In the hippocampus, Cre is exclusively expressed in the immature neurons in the hippocampal dentate gyrus from postnatal day 5 (P5) onward^48^. Gene recombination occurs in the cre-expressing immature neurons born at P5 and afterwards. Thus, that is why NICD is overexpressed in both immature and mature granular neurons in the adult hippocampus shown in Supplemental Fig. 3.

Gross anatomical analysis with cresyl violet staining did not show any global brain malformation in Notch1-cKO mice and NICD-cOE mice compared to control mice (Supplemental Fig. 4a–e). Since Notch1 expression was specifically interrupted and NICD was over-expressed in DG granular neurons, we further determined the architecture of the DG in Notch1-cKO, NICD-cOE, and control mice. We found that the DG did not exhibit abnormal morphologies and that measurements of cell density in the GCL of DG did not show any significant difference compared with control mice (Supplemental Fig. 4f). These results suggested that conditional knockout of *notch1* or overexpression of *NICD* in DG granular neurons does not significantly change the architecture of the DG, or cause massive granular neuron death in the DG.

We then assessed the dendrite complexity of immature neurons in the adult hippocampus of Notch1-cKO mice and Notch1^flox/flox^ control mice by assessing dendritic arborization based on Dcx immunostaining (Fig. 3b–e). We measured by counting the total number of dendrites and then divided by the total number of immature neuron bodies. We obtained an average number of dendrite branches in each immature neuron. The average number of dendrite branches of each immature neuron was 2.76 ± 0.13 in the Notch1^flox/flox^ control mice (Fig. 3f); Notch 1 cKO reduced the average number of dendrite branches of each immature neurons to 1.39 ± 0.19 (p < 0.05) (Fig. 3f). In contrast, overexpression of NICD increased the number to 3.69 ± 0.39 (P < 0.01) (Fig. 4f). This result indicated that Notch1 is required for dendrite branching, confirming the conclusion using the Notch1 single cell knockout we reported above.

### Conditional knockout of Notch1 reduced mTOR signaling pathway in the immature neurons in the adult hippocampus

**Figure 5:**
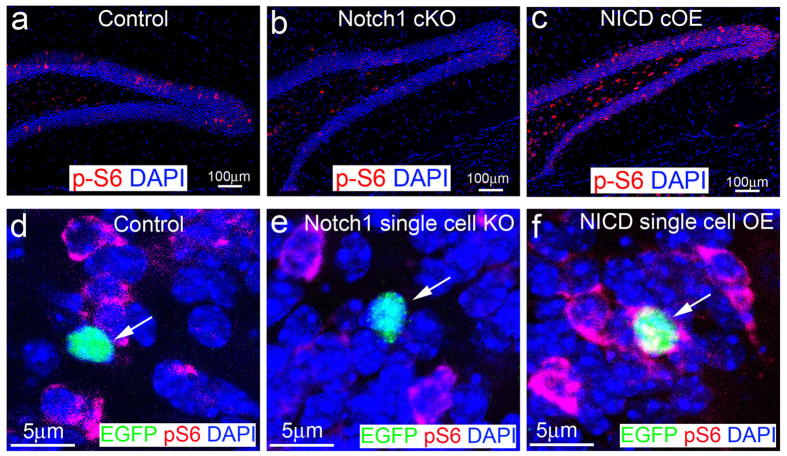
mTOR activity was affected by altered Notch1 or Notch intracellular domain (NICD) expression. (**a–c**) The pS6, a maker for mTOR signal pathway activation, expression (red) in control, Notch1-conditional knockout (cKO) or NICD-conditional overexpression (cOE) mouse hippocampus respectively. (**d–f**) The pS6 expression (red) in Cre-GFP infected cells of the olfactory bulb in wild type, Notch1 floxed, or NICD floxed mice respectively. *notch1* was knocked out or NICD was overexpressed in the GFP-positive cell of Notch1 floxed or NICD floxed mice respectively.

To explore the molecular mechanism that Notch1 signal regulates morphological development of immature neurons, we assessed the mTOR signaling activity. One of the well-studied targets of mTOR kinase is ribosome S6 kinase 1, which, in turn, phosphorylates ribosomal protein S6 (RPS6)^49^,^50^,^51^. Thus, the antibody to phosphorylated ribosomal protein S6 (p-S6) is widely used to monitor the activity of the mTOR pathway^51^. p-S6 was maintained at a very low expression level in the adult hippocampus (Fig. 5a). We found that p-S6 expression was significantly reduced in the adult hippocampus of the Notch1 cKO mice (Fig. 5b). In contrast, it was strongly increased in the adult hippocampus of NICD cOE mice (Fig. 5c). These data suggested that disrupted or activated Notch1 signaling altered mTOR signaling activity in the hippocampal granular neurons.

To further confirm that the Notch1 pathway regulates mTOR signal activity, we conditionally knocked out Notch1 or over-expressed NICD in the adult born immature neurons in the olfactory bulb, another region that has neurogenesis in the adult animal. We injected retroviruses expressing Cre and enhanced green fluorescent protein (EGFP) into the subventricular zone of wild type, Notch1^flox/flox^, and flox-stop-flox-NICD transgenic mice at the age of 12 weeks old. The subventricular zone is another area that has NPCs. Similar to the postnatal neurogenesis in the hippocampus, the retrovirus infected neural precursors, migrated into the olfactory bulb, and differentiated into new neurons. We did not detect pS6 immunoactivity in the wild type adult-born neurons with EGFP expression (Fig. 5d, identified by the white arrow), or in the Notch1 mutant adult-born neurons (Fig. 5e, identified by the white arrow) in the olfactory bulb. Nonetheless, the pS6 immunoactivity in the NICD over-expressed adult-born neurons (Fig. 5f) was significantly increased 3 weeks after viral injection. These data suggested that the Notch pathway regulates mTOR signaling in the neurons in the postnatal brain. The activity of mTOR signaling has been shown to play a critically important role in regulating dendrite arborization^49^,^52^. These data may suggest a possibility that the Notch1 pathway controls dendrite arborization through regulating mTOR signaling.

### Conditional knockout of Notch1 impaired spatial memory

Adult-born neurons in the hippocampus play roles in learning and memory^53^,^54^. In order to determine whether the disruption of Notch1 signaling affects behavior, we assessed the spatial learning and memory of Notch1 cKO mice (n = 10), NICD cOE mice (n = 10), and control mice (n = 13) with the Morris water maze (MWM) test. This test measures the amount of time it takes for a mouse to escape from the water and to find a platform hidden in the pool of water (escape latency).

**Figure 6:**
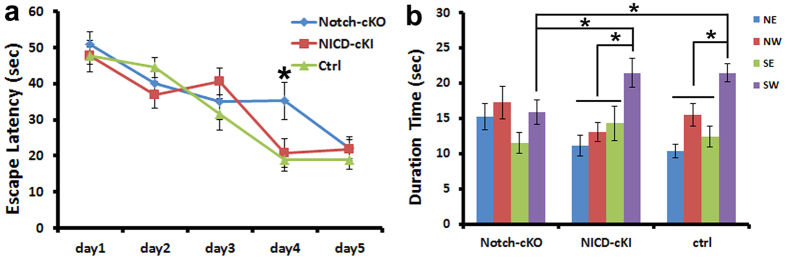
Conditional knocked out Notch1 in newborn neurons of the hippocampus impaired spatial learning and memory with the Morris water maze (MWM) test. (**a**) The 5 days learning trial of Notch1 conditional knockout (cKO), Notch intracellular domain (NICD) conditional overexpression (cOE), and control mice. At day 4, Notch1 cKO mice showed a need for a significantly longer time to find the hidden platform compared to NICD cOE and control group mice. (**b**) Probe test result of Notch1 cKO, NICD cOE and control mice. Notch1 cKO mice spent less time in the target quadrant than the NICD cOE and control group mice. (n = 10 for Notch1 cKO and NICD cOE mice, n = 13 for control mice, *p < 0.05).

On the first training day, the escape latency of each group was not significantly different, which ensured that the mice began the test at the same performance level. The escape latency of the control group reduced each day in the first 4 days and reached the best time on the 5^th^ day (Fig. 6a). For those Notch1 cKO mice, their escape latencies were also slowly reduced in the first 3 days of training, but still showed a significantly longer latency, even after 4 days of training (32.25 ± 5.11 sec vs. control 18.94 ± 3.08 sec, p < 0.05, Fig. 6a). In contrast, for those NICD cOE mice, their escape latencies were not different compared to the control group (20.78 ± 3.91 sec vs. control 18.94 ± 3.08 sec, p > 0.05, Fig. 6a). These data indicated that Notch1-cKO caused a delay in escape latency at the 4^th^ day of the training, while NICD overexpression did not obviously affect learning with the Water-maze test.

Following 5 days of training, a probe-trial test was then conducted to assess the reference memory. In the probe test, control mice showed a preference for staying in the target quadrant of the pre-existing platform, which was indicated with the more than 35.79% of latency in the target quadrant (35.79 ± 2.12%) (Fig. 6b). Notch1 cKO mice stayed for less time in the target quadrant (26.53 ± 2.82%, p < 0.05, versus control group) (Fig. 6b), suggesting that their memory about the position of the pre-existing platform was impaired. NICD cOE mice had a similar pattern compared to control mice (35.78 ± 3.74%, p > 0.05); they stayed in the target quadrant a significantly longer time. These data indicated that conditionally disrupting Notch1 signaling in the adult-born neurons in the hippocampus impaired spatial memory.

## Discussion

Notch1 is a receptor that is highly expressed in neural stem cells during early neural development and post-mitotic neurons in the postnatal brain^38^,^55^,^56^. Notch1 signaling is well known to play critically important roles in NSC fate determination during early neural development^11^,^12^,^57^,^58^; however, surprisingly little is known about its roles in neurons of the postnatal brain. A recent paper showed that conditionally knocking out Notch1 in the neural stem cells beginning from early neural development regulated cell fate and affected dendrite morphology of newborn neurons^59^. Nonetheless, it is still not known whether Notch1 directly regulates dendrite morphology or affects dendrite development indirectly due to its effect on fate alternation. In this study, we conditionally knocked out Notch1 specifically in the postmitotic neurons in the postnatal brain without affecting neural stem cell fate. We showed that Notch1 signaling directly regulates dendrite morphogenesis in the postmitotic neurons. Conditional knockout of Notch1 in the adult-born immature neurons in the adult hippocampus reduced the number of dendrite branches. In contrast, NICD overexpression significantly increased the average number of dendrite branches in each adult-born neurons. Retrovirus-mediated single cell knockout of Notch1 or overexpression of NICD confirm the results and indicate that the effect of Notch1 signaling on dendrite development is cell autonomous events. We further found that mTOR signaling might mediate Notch1 modulation of dendrite arborization.

The components of the Notch signaling pathway appear to be highly conserved among species^1^,^2^. The Notch receptor acts as a membrane-bound transcription factor that is released to the nucleus by a two-step intramembrane proteolysis^60^. When ligands (Jagged1, Jagged2, Delta1, Delta-like 1 [Dll1], Dll3, and Dll4 in mammals or Delta and Serrate in *Drosophila*) bind to Notch receptors (Notch1, Notch2, Notch3, and Notch4 in mammals and Notch in *Drosophila*), it initiates the cleavage at a position 12 amino acids from the intracellular membrane by metalloproteases of the ADAM family. The truncated Notch renders further cleavage by a γ-secretase-dependent protease to release the Notch intracellular domain (NICD)^3^,^4^,^5^,^6^. NICD subsequently translocates to the nucleus, and activates the major downstream nuclear target for Notch^1^,^61^,^62^,^63^. Presenilin (PS) plays a role in γ-secretase complex. Mutations in presenilins is a key factor in familial Alzheimer’s disease (FAD)^64^,^65^. Notch1 and amyloid precursor protein (APP) are competitive substrates for presenilin1-dependent gamma-secretase cleavage^66^,^67^.

Amyloid ß-protein precursor (APP), like Notch, is a type I transmembrane protein, and its sequential proteolysis by ß-secretase and gamma-secretase generates the Aß40 and Aß42 peptides, which are made normally throughout life but can accumulate abnormally in Alzheimer’s disease^68^. A link between Notch and presenilins was first uncovered by Greenwald and colleagues, who carried out genetic screens designed to identify interacting genes with the LIN12/Notch receptor in C. elegans^69^. Subsequent analyses implicated, as mentioned above, the presenilin complex in the intracellular, signal-producing cleavage of the Notch receptor. The FAD-associated mutations in presenilins affect Notch cleavage and, consequently, Notch function^70^. In this regard, it is also interesting that Notch pathway elements are expressed in neurons of the adult cerebral cortex and that Notch signaling regulates postmitotic neuronal differentiation and neuronal size (see above). Notch expression (but not necessarily pathway activity) is reportedly upregulated in the brains of individuals with Alzheimer’s disease^38^, and Notch1 and presenilins seem to co-localize in neurons in adults. Moreover, in cultured hippocampal neurons, transfection of FAD-linked presenilin variants interferes with nuclear translocation of Notch^71^, whereas mice lacking presenilin in the forebrain have impaired memory and develop age-dependent neurodegeneration^72^,^73^. Inhibitors of gamma-secretase have been developed as possible therapeutic strategies for AD. Interestingly, the majority of these inhibitors also down regulate Notch signaling and impair Notch function^74^,^75^,^76^ Even gamma-secretase inhibitors that could specifically target APP cleavage would result in diminished generation of the APP intracellular domain and could alter Notch signaling^77^. These data suggest a link in intramembrane proteolysis and function between Notch signaling and APP. However, it is not known if any of the pathologies associated with Alzheimer’s disease are caused by the malfunction of the Notch pathway.

Notch1 is expressed at low levels throughout the cortex and its level of expression is particularly high in the lateral subventricular zone (SVZ), subgranular zone (SGZ) of the hippocampus, and cerebellum^55^. There is continuous neurogenesis in the SVZ and SGZ thoughout the life span of mammalian animals^16^,^18^,^78^,^79^,^80^. Adult-born neurons have been shown to be able to integrate in the neural circuitry in the hippocampus and play a significant role in learning and memory. *In vitro* experiments indicate that Notch1 may play a role in postmitotic neurons by regulating neurite formation^37^,^81^,^82^,^83^. Our data showed that Notch1 plays a critical role in dendrite arborization of adult-born neurons. Since dendrites provide enormous surface area for spine formation and determine the range and scope of synaptic inputs, dendritic degeneration could likely cause significant disruption in synaptic transmission between neurons, in turn, contributing to functional deficits. Notch1 regulates hippocampal plasticity^84^. A constitutive decrease in Notch signaling (heterozygous notch knockout) can result in specific learning and memory deficits^8^. This study further shows that conditional knockout *notch1* reduces dendrite arborization of the adult-born neurons in the hippocampus and causes impairment in learning and memory. These results suggest that PS1 mutation may affect both APP degradation and Notch1 signaling. Abnormalities in Notch-dependent transcription may impair development of adult-born neurons in the hippocampus and contribute to the cognitive deficits associated with Alzheimer’s disease and Alagille and CADASIL syndromes.

## Additional Information

**How to cite this article**: Ding, X.-F. *et al*. Postnatal dysregulation of Notch signal disrupts dendrite development of adult-born neurons in the hippocampus and contributes to memory impairment. *Sci. Rep*. **6**, 25780; doi: 10.1038/srep25780 (2016).

## Supplementary Material

Supplementary Information
