# Supplementary material for: Postnatal dysregulation of Notch signal disrupts dendrite development of adult-born neurons in the hippocampus and contributes to memory impairment
Source: Sci Rep. 2016 May 13;6:25780. doi: 10.1038/srep25780 (PMC4865733; doi:10.1038/srep25780)
Supplement: Supplementary Information [file srep25780-s1.pdf]

Postnatal dysregulation of Notch signal disrupts dendrite development of adult-born neurons in the hippocampus and contributes to memory impairment

Xue-Feng Ding[1, 2], Xiang Gao[1], Xin-Chun Ding[3], Ming Fan[2], Jinhui Chen[1*]

[1] Spinal Cord and Brain Injury Research Group, Stark Neuroscience Research Institute, and Department of Neurological Surgery, Indiana University School of Medicine, Indianapolis, IN 46202, USA

[2] Department of Cognitive sciences, Beijing Institute of Basic Medical Sciences, Beijing 100850, P. R. China.

[3] Department of Pathology, Indiana University School of Medicine, Indianapolis, IN 46202, USA

***Corresponding Author:**

Spinal Cord and Brain Injury Research Group,

Stark Neuroscience Research Institute & Department of Neurological Surgery,

Indiana University School of Medicine,

320 W. 15th Street, Indiana University, Indianapolis, IN 46202, USA.

Phone: +1 317 278 5782

Fax: +1 317 278 5849.

E-mail address: chen204@iupui.edu.

Supplemental Figures and Legends

Suppl. Figure 1

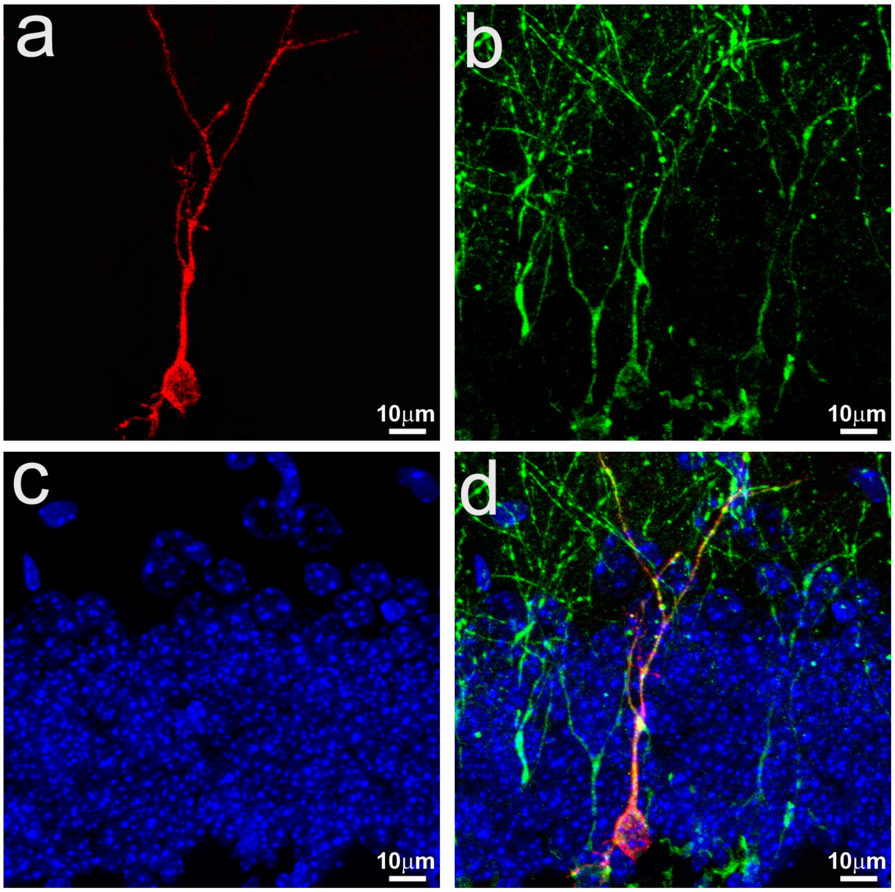

Supplemental Figure 2

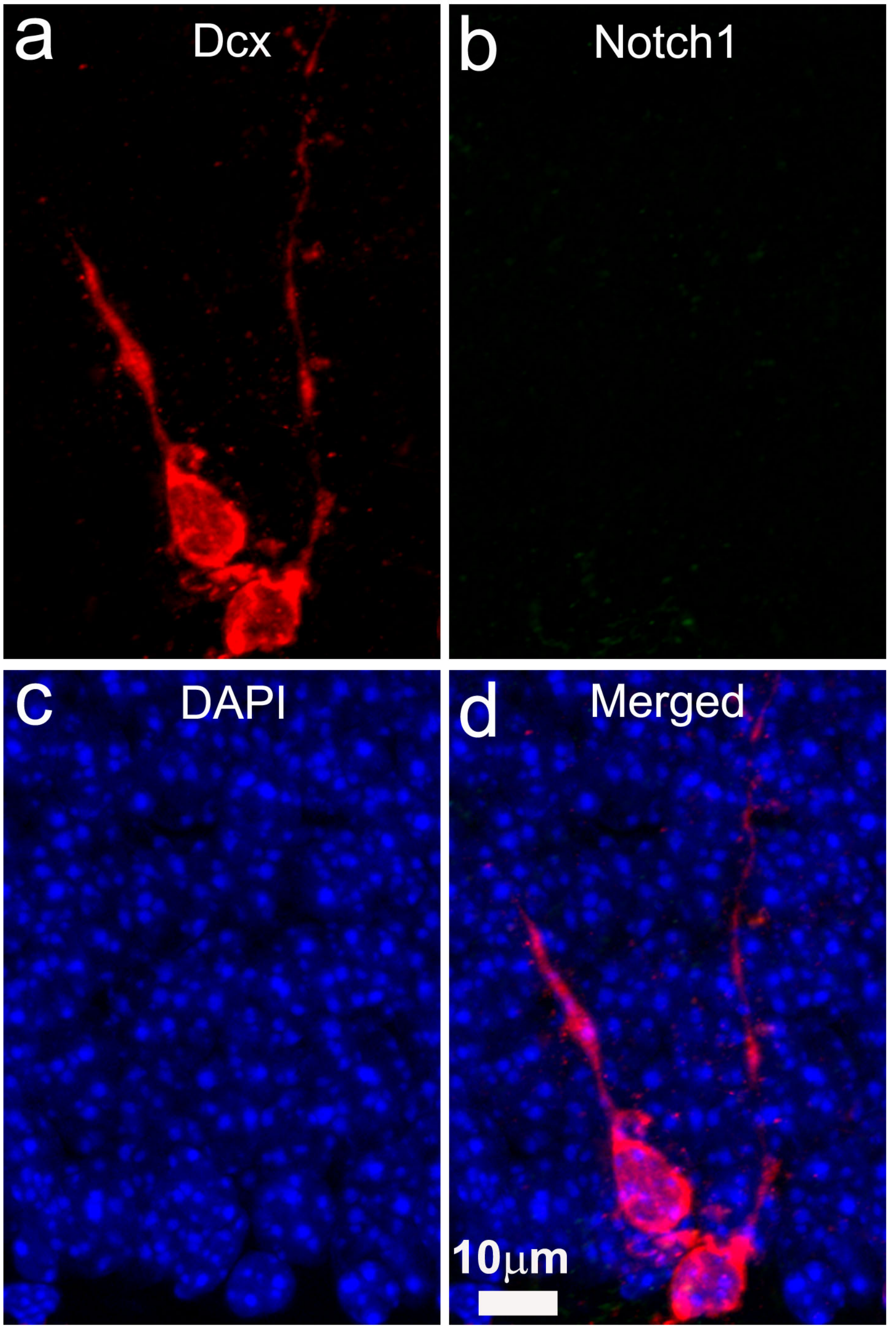

Supplemental Figure 3

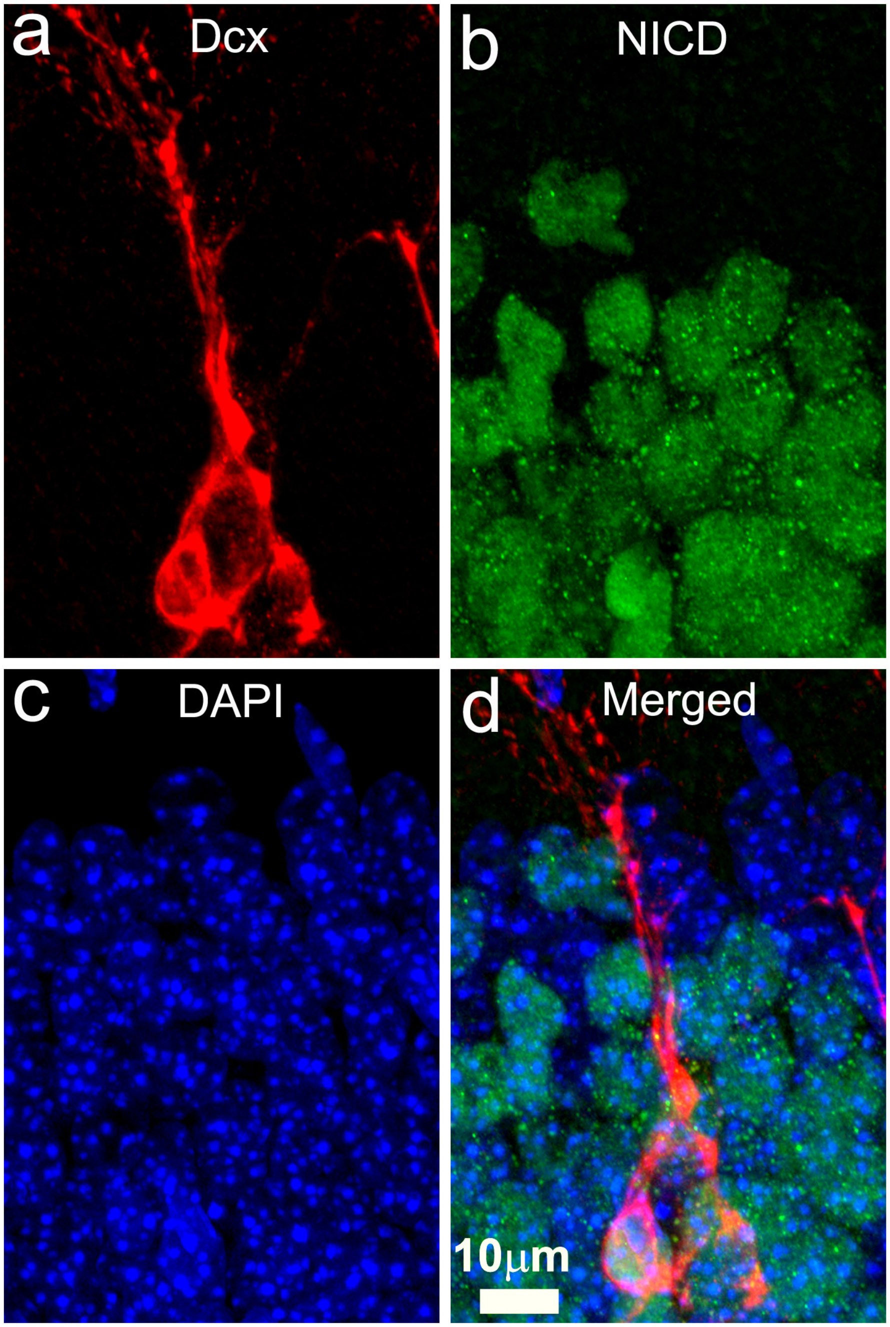

Supplemental Figure 4

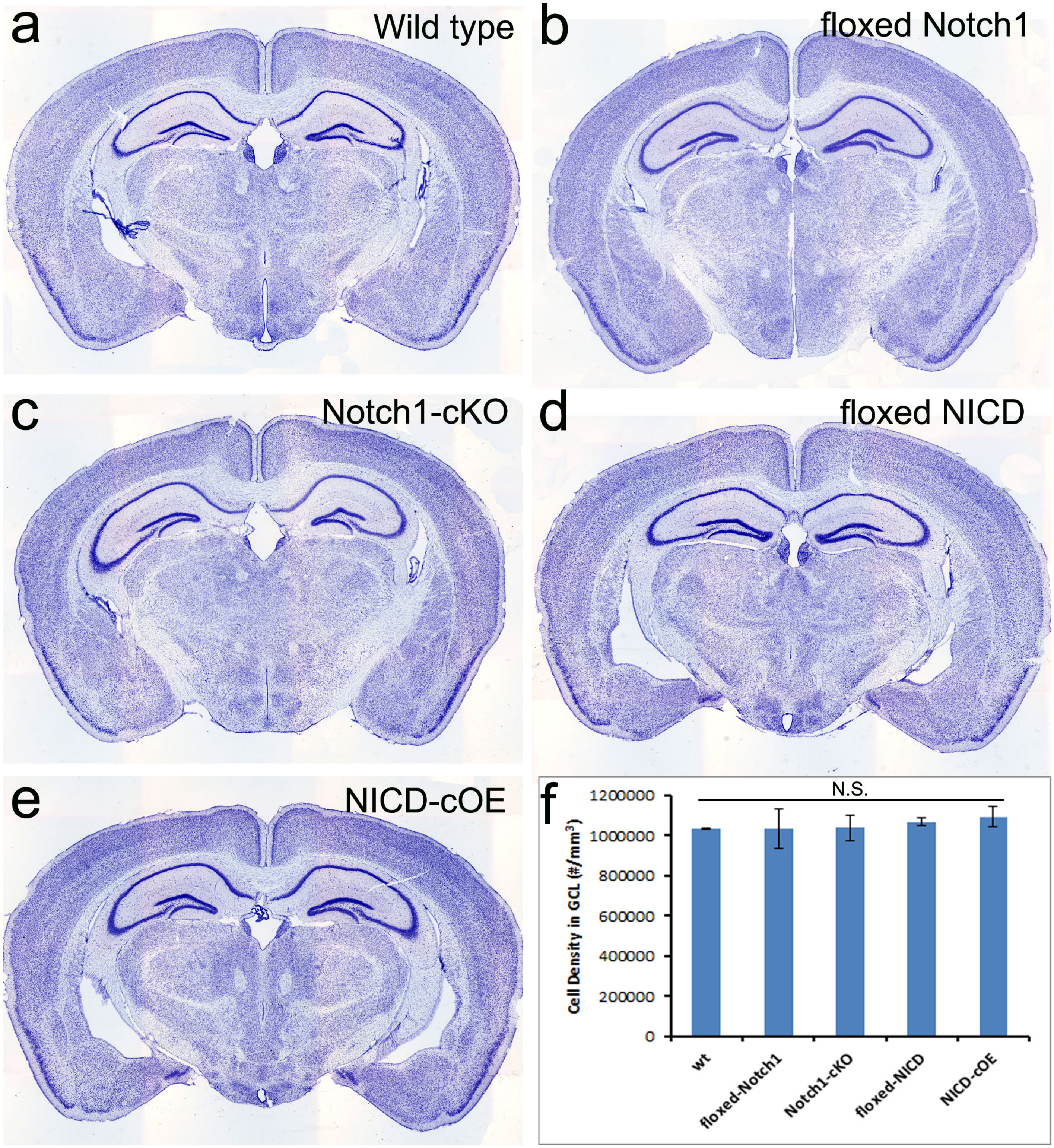

Supplemental Figure legends

Supplemental. Figure 1. 3 weeks after retrovirus infection, red fluorescent protein (RFP)-positive cell in hippocampal dentate gyrus co-labeled with doublecortin (Dcx), a marker for immature neurons. a. RFP labeled cell in the granular cell layer (GCL) of the dentate gyrus (DG; red). b. Dcx staining of DG (green). c. DAPI staining showed the structure of DG. d. Merged image of a-c demonstrated the co-labeling of RFP and Dcx in retrovirus infected newborn immature neuron.

Supplemental. Figure 2. Notch1 expression is undetectable in doublecortin (Dcx)-positive immature neurons in hippocampus of Notch1 conditional knockout (cKO) mice. a. Dcx staining (red). b. Notch1 staining (green). c. DAPI staining showed the granular cell layer (GCL) in the dentate gyrus (DG). d. merged image of a-c. There is no detectable Notch1 expression in Dcx-positive cells.

Supplemental. Figure 3. Notch intracellular domain (NICD) expression is dramatically increased in doublecortin (Dcx)-positive immature neurons and mature neurons in hippocampus of Notch intracellular domain (NICD) conditional overexpression (cOE) mice. a. Dcx staining (red). b. NICD staining (green). c. DAPI staining showing the granular cell layer (GCL) in the dentate gyrus (DG). d. merged image of a-c.

Supplemental. Figure 4. Brain gross structure and granular cell layer (GCL) cell density of Notch1 conditional knockout (cKO), Notch intracellular domain (NICD) conditional overexpression (cOE), and control mice. a-e. Nissl staining of tissue sections showed the brain gross structure of wild type, Notch1 floxed, Notch1-cKO, NICD floxed, and NICD-cOE mice respectively. f. GCL cell density in five different groups and there is no significant difference between the groups. n=5 for each group. N.S.: not significant.
